# Projections of the Mouse Primary Visual Cortex

**DOI:** 10.3389/fncir.2021.751331

**Published:** 2021-11-19

**Authors:** Arbora Resulaj

**Affiliations:** ^1^Department of Biology, University of Toronto Mississauga, Mississauga, ON, Canada; ^2^Department of Cell and Systems Biology, University of Toronto, Toronto, ON, Canada

**Keywords:** primary visual cortex (V1), projections, mouse, synaptic connectivity, behavior

## Abstract

Lesion or damage to the primary visual cortex (V1) results in a profound loss of visual perception in humans. Similarly, in mice, optogenetic silencing of V1 profoundly impairs discrimination of orientated gratings. V1 is thought to have such a critical role in perception in part due to its position in the visual processing hierarchy. It is the first brain area in the neocortex to receive visual input, and it distributes this information to more than 18 brain areas. Here I review recent advances in our understanding of the organization and function of the V1 projections in the mouse. This progress is in part due to new anatomical and viral techniques that allow for efficient labeling of projection neurons. In the final part of the review, I conclude by highlighting challenges and opportunities for future research.

## Introduction

V1 is the first cortical area to receive visual input (Felleman and Van Essen, 1991; Siegle et al., 2021). It performs numerous computations locally (for a review, see Froudarakis et al., 2019; Katzner et al., 2019; Niell and Scanziani, 2021), and it distributes information to more than 18 brain areas (Oh et al., 2014; Zhang et al., 2016; Han et al., 2018; Harris et al., 2019). These projections to the different brain areas are thought to play distinct roles: projections to higher visual cortical areas (Wang and Burkhalter, 2007) are thought to extract visual features that match the features represented by higher visual cortical areas (for a review see Glickfeld and Olsen, 2017), projections to the thalamus are thought to provide alternative routes of communication between the cortical areas (secondary thalamus: Guillery and Sherman, 2002; Blot et al., 2021), and projections to major subcortical areas like the superior colliculus, striatum, or the brainstem nuclei, are thought to modulate simple or innate behaviors (Khibnik et al., 2014; Zhao et al., 2014; Liang et al., 2015; Liu et al., 2016; Ruediger and Scanziani, 2020; Tang and Higley, 2020).

This review focuses on recent studies that reveal, or have started to reveal, general principles about V1 projections: whether the different V1 projections are anatomically segregated, whether V1 projections send specialized information to different target areas, and whether we can pinpoint a distinct behavioral role to a unique V1 projection.

## Organization of V1 Projections

**FIGURE 1:**
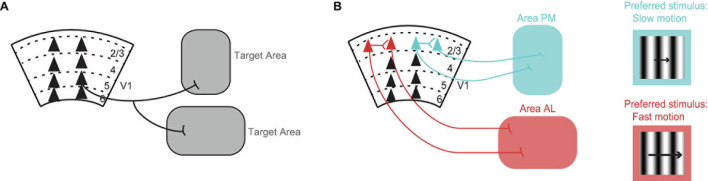
Organization of V1 projections. **(A)** Axons of most V1 neurons target multiple brain areas (Han et al., 2018). Triangles represent cell bodies, lines indicate axons. Numbers and dashed lines mark the different layers of V1. **(B)** V1 neurons projecting to visual cortical area PM (blue) and V1 neurons projecting to visual cortical area AL (red) avoid making local connections with each other within V1 (Kim et al., 2018). Conventions as in A. V1 neurons projecting to area PM (blue) prefer, i.e., fire the most spikes for, the slower moving stimuli, while V1 neurons projecting to area AL (red) prefer faster moving stimuli (Kim et al., 2018). The same preference is seen in V1 axons projecting to these areas from layer 5 (Glickfeld et al., 2013a). The drifting grating represents the visual stimulus. The length of the arrow is proportional to speed.

Cortical neurons have axon collaterals that may terminate in different brain areas. In other words, we expect that some V1 neurons will target multiple brain areas. Yet, how common it is for V1 neurons to target multiple areas was not known until Han et al. (2018) showed that in fact most V1 neurons target more than one area (Figure 1A). This was achieved by using single cell electroporation of a GFP-encoding plasmid combined with whole brain fluorescence imaging, or in separate experiments, by using MAPseq where unique DNA barcodes are taken up by V1 neurons and transported to the axons allowing for subsequent DNA sequencing of the target area. By focusing the analysis on V1 projections to higher visual cortical areas, the authors found that there were four sets of areas that received more shared axons than would be expected by chance when considering the probability of a V1 neuron projecting to each area (i.e., when multiplying the projection probabilities to each individual area). The four sets of areas were: posteromedial (PM) and anteromedial (AM) areas, lateromedial (LM) and anterolateral (AL) areas, PM and LM and laterointermediate (LI) areas, and PM and AM and rostrolateral (RL) areas. Therefore, although most V1 axons target more than one area, the axonal branches preferentially target a subset of areas (consistent with Berezovskii et al., 2011).

Cortical neurons that target different areas also form local connections with each other within the area of origin (Brown and Hestrin, 2009; Cotel et al., 2018; Kim et al., 2018). These connections can influence whether neurons send similar or different information to their respective target areas. Kim et al. (2018) looked at the connection probability in V1 between neurons projecting to higher visual cortical area AL and neurons projecting to higher visual cortical area PM. To label V1 neurons according to their target area, the authors injected retrograde tracers of different colors in the two different target areas. By performing *in vitro* electrophysiology, they found that pairs of neurons, in which each neuron targeted a different area, had a highly reduced connection probability with each other compared to chance (Cossell et al., 2015; Kim et al., 2018). Hence, V1 neurons projecting to area AL and V1 neurons projecting to area PM avoid making local connections with each other within V1 and thus they are segregated from each other (Figure 1B).

A contrasting study by Brown and Hestrin (2009) showed that neurons projecting to the same area form enriched local connections with each other. By using retrograde tracers of different colors and *in vitro* electrophysiology, the authors found that neurons that project to the striatum have a much higher probability of connecting to each other than connecting to other neurons in V1. Consistent with this higher connection rate, Lur et al. (2016) found that the activity of individual V1 neurons projecting to the striatum is more correlated with the activity of neurons projecting to the striatum than with the activity of neurons projecting to other areas. What might be the function of these enriched interconnections between neurons projecting to the same area? One possibility is that they amplify cortical signals which may be required to achieve depolarization of striatal neurons downstream (Brown and Hestrin, 2009). Another argument for the observed pattern of local connections is the optimization of wiring length (Chklovskii and Koulakov, 2004).

Lastly, V1 neurons that target different areas not only form local connections with each other within V1, but they also receive feedback from these areas that they target. By using subcellular channelrhodopsin-2-assisted circuit mapping, Young et al. (2021) found that the feedback inputs into V1 neurons in deep layers were stronger if the contacted V1 neuron also targeted that particular area, thus demonstrating the specificity of the feedback input for the different V1 projections (consistent with Kim et al., 2015). Although the impact of feedback on V1 activity is likely not strong (Goldbach et al., 2021), feedback loops may be important for selective visual attention (Zhang et al., 2014).

## Information in the V1 Projections

V1 sends projections to numerous brain areas and these areas are thought to have distinct functions. For example, the lateral higher visual cortical areas are associated with the ventral (what) stream for object recognition, while the anterior and medial areas are associated with the dorsal (where) stream for movement information (Wang et al., 2012; Saleem, 2020), as proposed in primates (Goodale and Milner, 1992). A major question has been whether V1 sends the same unspecific information to all its target areas or whether it sends specific and specialized information to each target area. Several studies have now demonstrated that V1 sends specific information to some of the higher visual cortical areas (Jarosiewicz et al., 2012; Glickfeld et al., 2013a; Matsui and Ohki, 2013; Rasmussen et al., 2020; Blot et al., 2021; for an exception see Murgas et al., 2020) and to subcortical areas (Kim et al., 2015; Lur et al., 2016; Tang and Higley, 2020).

An early example showing that V1 sends specific information to some of the higher visual areas comes from Glickfeld et al. (2013a). By using 2-photon calcium imaging of V1 axons terminating in either higher visual cortical area AL or higher visual cortical area PM, Glickfeld et al. (2013a) found that the visual responses to drifting gratings were different in the different areas. The V1 axons terminating in area AL responded best to faster moving stimuli while axons terminating in area PM responded best to slower moving stimuli (Glickfeld et al., 2013a). The same preference for speed had been shown earlier for neurons in area AL and neurons in area PM (Andermann et al., 2011; Marshel et al., 2011; Roth et al., 2012). Hence, V1 axons send specialized information to each of these areas and, further, this information matches the responses of the recipient neurons. It is tempting to conclude that V1 confers these preferences for speed to these areas, however, Tohmi et al. (2014) showed that the speed preference in these areas remains after ablation of V1. Instead, it is the lesion of the superior colliculus that eliminates the differences in the visual properties between these areas (Tohmi et al., 2014). Yet, V1 might confer other visual properties to the higher visual areas, for example the spatial modulation of visual responses (Diamanti et al., 2021).

Another example showing that V1 sends specific information to different subcortical areas comes from Tang and Higley (2020). This study used a visually cued eye blink conditioning task and 2-photon calcium imaging of V1 neurons projecting to either the pons or to the striatum. The authors found that neurons projecting to the pons had larger responses for correct eye blink responses versus incorrect eye blink responses, while neurons projecting to the striatum did not show a difference. Thus, Tang and Higley (2020) provide more evidence that V1 sends specific information to some of its target areas. Furthermore, it suggests the interesting possibility that V1 output may directly influence motor related areas because action-related signals are already present in these V1 projections, consistent with previous studies showing responses in V1 that correlate with the timing of action (Namboodiri et al., 2015).

The studies above show that V1 routes specific information to its target areas. A related question is whether the different projections are segregated from each other and whether information in these projections can be separately modulated, for example, by inputs into V1. By comparing wild type mice with *Frmd7*^TM^ mutant mice which have a disruption in the direction selectivity in the horizontal motion in the retina, Rasmussen et al. (2020) found that V1 neurons projecting to higher visual cortical area RL showed a disruption in the horizontal direction selectivity while neurons projection to higher visual cortical area PM did not show a disruption. Therefore, information in the different projections can be separately modulated (consistent with Huh et al., 2018).

## Role of V1 Projections During Behavior

Over the last decade, several studies have identified visual tasks for mice that critically depend on V1 (Poort et al., 2015; Goard et al., 2016; Resulaj et al., 2018). By optogenetically silencing V1 output during these tasks, these studies have shown that performance accuracy drops to near chance levels. Yet, it is important to note that lesioning V1 has yielded mixed results: for discrimination of oriented gratings, lesioning V1 dropped accuracy to chance levels in Resulaj et al. (2018) but not in Prusky and Douglas (2004). One possibility is that, in the study of Prusky and Douglas (2004), mice might have re-learned to perform the task using orientation information from the superior colliculus (Feinberg and Meister, 2015). Several studies have also identified visual tasks where V1 only plays a modulatory role (Glickfeld et al., 2013b; Ruediger and Scanziani, 2020). In these tasks, V1 is thought to affect behavior by modulating the activity of subcortical structures (Zhao et al., 2014; Ruediger and Scanziani, 2020).

Can we pinpoint a distinct behavioral role to a specific V1 projection? Two recent studies have provided direct evidence for distinct roles (Ruediger and Scanziani, 2020; Tang and Higley, 2020). Tang and Higley (2020) showed that optogenetically suppressing V1 neurons projecting to the pons impaired a visually cued eye blink conditioning response, while suppressing V1 neurons projecting to the striatum had no effect. Another example showing distinct roles in behavior for different V1 projections comes from Ruediger and Scanziani (2020), where the authors permanently ablated either V1 neurons projecting to the striatum or V1 neurons projecting to the superior colliculus during a visual detection task. The authors found that V1 neurons projecting to the striatum controlled the speed at which mice learned the task, while V1 neurons projecting to the superior colliculus did not affect the speed of learning but affected the sensitivity of detecting the stimulus once the task was learned. Permanent ablation and acute optogenetic silencing have been shown to have different effects on downstream targets (Otchy et al., 2015) and therefore these two methods can be used as complementary approaches when assessing the role of specific projections.

## Future Directions

Different V1 projections will most certainly have different roles in the target areas, and some roles will likely only be understood in the context of behavior. Diverse visual tasks that depend on V1 have been developed (Poort et al., 2015; Goard et al., 2016; Resulaj et al., 2018). These tasks, in combination with controlled perturbations (Carrillo-Reid et al., 2019; Marshel et al., 2019) and recordings of neural activity (Jun et al., 2017; Steinmetz et al., 2019; Stringer et al., 2019a, b), promise to reveal how V1 plays such a critical role in visual perception.

**FIGURE 2:**
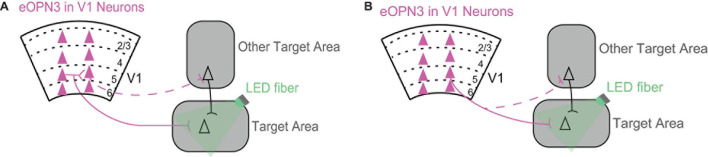
Using inhibition of neurotransmitter release from axonal terminals to dissect the circuits through which V1 mediates its effects in the target area. **(A)** A V1 projection can mediate its effects in the target area either directly *via* its projection to the target area (solid line) or indirectly *via* its local connections with other V1 projections (dashed line). Triangles represent cell bodies, lines indicate axons. Scarlet indicates expression of eOPN3 in V1 neurons. eOPN3 is a mosquito derived rhodopsin that inhibits neurotransmitter release when axon terminals are illuminated with light (here, green LED fiber; Mahn et al., 2021). For a similar rhodopsin see Copits et al. (2021). **(B)** A V1 projection can mediate its effects in the target area either directly *via* its axonal branch to the target area (solid line) or indirectly through another axonal branch targeting another area (dashed line). Conventions as in **(A)**.

One exciting future direction is to reveal the role of direct V1 projections in a particular target area. This is now possible thanks to a recent tool, inhibitory opsins that suppress neurotransmitter synaptic transmission (Copits et al., 2021; Mahn et al., 2021). To reveal the contribution of the direct V1 projection to the target area, it is now possible to shine light at the direct axonal branch in the target area to selectively suppress neurotransmitter release only in this area (Figure 2). Different V1 projections make overlapping local connections with each other within V1. Therefore, a particular V1 projection can mediate its effects either directly *via* its direct projection to the target area or indirectly *via* its local connections with other V1 projections (Figure 2A). In addition, V1 neurons have axons that branch out in multiple areas, which means they can further mediate their effects indirectly through another axonal branch targeting another area (Figure 2B).

However, a word of caution on the presumed role of V1 projections is worth mentioning. It is generally assumed that the role of V1 neurons is to carry visual information to the target areas. However, it is possible that the particular V1 projection is only providing unspecific excitation to the target area and that removal of this excitation leaves the downstream circuits in an unbalanced state and unable to function (for a cautionary example from the motor cortex, see Otchy et al., 2015). This possibility needs attention especially following recent demonstrations that visual information can reach specific target areas through alternate routes that do not involve V1, for example through the extrageniculate pathway *via* the superior colliculus (Tohmi et al., 2014; Beltramo and Scanziani, 2019). Is the visual information from V1 used in the target area? To address this, it will be important to record in the target area while perturbing the particular V1 projection. Remarkably, to perturb activity, it is now possible to use 2-photon guided single cell optogenetics and selectively activate a few neurons to recapitulate some of the pattern of V1 activity evoked by a visual stimulus and elicit the correct behavioral response (Carrillo-Reid et al., 2019; Marshel et al., 2019). These recently developed techniques are poised to advance our understanding of the organization and function of the numerous V1 projections.

## Author Contributions

The author confirms being the sole contributor of this work and has approved it for publication.

## Conflict of Interest

The author declares that the research was conducted in the absence of any commercial or financial relationships that could be construed as a potential conflict of interest.

## Publisher’s Note

All claims expressed in this article are solely those of the authors and do not necessarily represent those of their affiliated organizations, or those of the publisher, the editors and the reviewers. Any product that may be evaluated in this article, or claim that may be made by its manufacturer, is not guaranteed or endorsed by the publisher.
